# Host Cell Central Carbon Metabolism and Cellular NAD^+^ Pool Regulate Efficient Replication of Vesicular Stomatitis Virus

**DOI:** 10.3390/v18030326

**Published:** 2026-03-06

**Authors:** Kush K. Pandey, Bikash R. Sahoo, D. S. McVey, Asit K. Pattnaik

**Affiliations:** 1 School of Veterinary Medicine and Biomedical Sciences, University of Nebraska-Lincoln, Lincoln, NE 68583, USA; kpandey2@huskers.unl.edu (K.K.P.); dmcvey2@unl.edu (D.S.M.); 2 Nebraska Center for Virology, University of Nebraska-Lincoln, Lincoln, NE 68583, USA

**Keywords:** vesicular stomatitis virus, 2-DG, glycolysis, pentose phosphate pathway, glutaminolysis, NAD^+^ metabolism, siRNA-mediated depletion

## Abstract

Vesicular stomatitis virus (VSV) is a promising oncolytic virus whose replication efficiency and tumor selectivity are strongly influenced by host cell metabolism. Cancer cells, including glioblastoma, exhibit profound rewiring of central carbon metabolism to sustain proliferation, redox balance, and biosynthetic demand, yet how these metabolic states regulate VSV replication remains incompletely defined. Here, we investigated the dependency of VSV replication on glycolysis, the pentose phosphate pathway (PPP), and glutamine metabolism in A172 human glioblastoma cells. Pharmacologic inhibition of glycolysis using 2-DG strongly suppressed VSV replication in a dose-dependent manner, highlighting a robust requirement for glycolytic flux and downstream intermediates. While inhibiting the PPP with 6-AN, a nicotinamide adenine dinucleotide (NAD) analog, markedly impaired viral replication, D-ribose was unable to rescue the inhibition, indicating that nucleotide precursor limitation alone was insufficient to explain this effect. Interestingly, depletion of glucose 6-phosphate dehydrogenase (G6PD), a key enzyme in the PPP, resulted in significant enhancement of VSV replication. Restoration of viral replication by NAD^+^ precursors in the presence of 6-AN or suppression of replication by the NAMPT inhibitor FK866 suggested NAD^+^ availability as a critical determinant of VSV replication. Additionally, blockade of glutaminase activity with BPTES reduced viral replication, underscoring the importance of anaplerotic pathways in glioblastoma cells. Collectively, these findings demonstrate that VSV replication is tightly coupled to metabolic programs, particularly those governing energy production and NAD(P)H balance. This work provides a metabolic framework for optimizing oncolytic VSV therapies and suggests that metabolic interventions in cancer treatment may influence oncolytic virus efficacy.

## 1. Introduction

Vesicular stomatitis virus (VSV) is an enveloped, non-segmented, negative-sense RNA virus belonging to the family *Rhabdoviridae* and has emerged as one of the most extensively studied platforms for oncolytic virotherapy. Although VSV is naturally associated with vesicular disease in livestock and typically causes only mild, self-limiting infections in humans, several intrinsic features make it particularly attractive for both fundamental virology and cancer therapy. These include its exceptionally broad cellular tropism, rapid replication kinetics, genetically simple genome, and the absence of widespread pre-existing immunity in the human population [1,2,3]. Together, these characteristics have positioned VSV as a powerful experimental system for dissecting virus–host interactions and as a promising agent for the selective targeting of malignant cells.

The approximately 11 kb VSV genome encodes five structural proteins: the nucleocapsid protein (N), phosphoprotein (P), matrix protein (M), glycoprotein (G), and the large RNA-dependent RNA polymerase (L). These components assemble into the characteristic bullet-shaped virion, measuring ~185 × 75 nm [4]. The G protein mediates viral attachment and membrane fusion, confers broad tropism, and is essential for infectious progeny production. The N, P, and L proteins together form the viral ribonucleoprotein complex that drives transcription and genome replication in the host cell cytoplasm, while the M protein plays a central role in virion assembly and budding, in addition to modulating host gene expression. Efficient VSV replication is therefore highly dependent on host cellular machinery and resources, requiring extensive reprogramming of host metabolic pathways to support viral RNA synthesis, protein production, and virion assembly and release.

Cancer cells, including glioblastoma, undergo profound metabolic reprogramming to sustain uncontrolled proliferation and survival in nutrient- and oxygen-limited environments [5,6]. Hallmarks of this reprogramming include elevated aerobic glycolysis, increased glutamine utilization, and heightened reliance on redox-balancing pathways to maintain metabolic homeostasis. Central carbon metabolism—encompassing glycolysis, the tricarboxylic acid (TCA) cycle, and the pentose phosphate pathway (PPP)—plays a pivotal role in sustaining these processes by generating ATP, nucleotides, amino acids, and reducing equivalents such as NADPH required for both normal cellular function and pathological growth, including tumor progression and viral replication [7,8,9]. Glycolytic intermediates are diverted into the PPP to support nucleotide biosynthesis, antioxidant defense, and the generation of substrates for N-linked glycosylation, while glutaminolysis replenishes TCA cycle intermediates and sustains mitochondrial metabolism and redox balance [10].

Recent studies have increasingly highlighted the potential of targeting host metabolic pathways to enhance the efficacy of oncolytic virotherapy. Perturbation of glycolysis, glutamine metabolism, or redox pathways has been shown to influence viral replication, tumor selectivity, and therapeutic outcomes in multiple oncolytic virus platforms [11,12,13,14,15]. Despite early observations demonstrating that inhibition of glycolysis with 2-deoxy-D-glucose (2-DG) suppresses VSV replication [16,17], a substantial knowledge gap remains in understanding how distinct branches of central carbon metabolism differentially regulate VSV replication in normal versus metabolically reprogrammed cancer cells.

Studying VSV replication in glioblastoma cells under conditions of targeted metabolic inhibition offers a unique opportunity to define how tumor-specific metabolic states shape oncolytic virus biology. Because VSV replication is highly sensitive to cellular energy availability, redox balance, and nucleotide pools, perturbation of central carbon metabolic pathways can reveal critical host determinants that either support or constrain viral replication. Understanding these relationships is essential for the rational integration of metabolic therapies with oncolytic virotherapy. Moreover, defining how glutamine-dependent metabolism in glioblastoma cells influences VSV replication has direct implications for therapeutic design. If VSV preferentially exploits metabolic features essential for tumor cell growth—such as elevated glycolytic flux or glutamine addiction—these dependencies may enhance tumor selectivity and therapeutic safety. Conversely, identifying metabolic constraints that limit viral replication may inform the development of optimized combination regimens that maximize oncolytic efficacy while minimizing toxicity.

In this study, we investigate the dependence of VSV replication on central carbon metabolism in A172 human glioblastoma cells, a metabolically active cell line well suited for mechanistic analysis of virus–host metabolic interactions. Using pharmacological inhibitors of glycolysis (2-DG), the oxidative PPP [6-aminonicotinamide (6-AN)], and glutaminase-mediated glutaminolysis [bis-2-(5-phenylacetamido-1,2,4-thiadiazol-2-yl) ethyl sulfide (BPTES)], we demonstrate that disruption of these metabolic pathways markedly suppresses VSV replication. While pharmacological inhibition of the PPP significantly dampened VSV replication, depletion of the key PPP enzyme glucose-6-phosphate dehydrogenase (G6PD) unexpectedly enhanced viral replication. Further mechanistic analyses identified NADPH-linked metabolic pathways and redox homeostasis as critical regulators of VSV replication, underscoring the importance of host redox metabolism in shaping virus–tumor cell interactions. Together, these findings provide a metabolic framework for optimizing VSV-based oncolytic strategies and deepen our understanding of how host cell metabolic states regulate VSV replication, pathogenesis, and therapeutic potential.

## 2. Materials and Methods

### 2.1. Cells and Viruses

A172 (ATCC CRL-1620) and Baby Hamster Kidney (BHK-21, ATCC CCL-10) cells were cultured in Dulbecco’s Modified Eagle’s Medium (DMEM) and Minimum Essential Medium (MEM), respectively. Both DMEM and MEM were supplemented with 10% heat-inactivated fetal bovine serum (FBS) and 1% antibiotic solution. Cells were maintained at 37 °C in a humidified incubator containing 5% CO_2_. VSV expressing enhanced green fluorescent protein (eGFP) fused to the P protein (VSV-PeGFP) was used for all infection experiments [18]. Plaque-forming assay was done to assay for infectious virus yield as described before [19]. Briefly, 100 µL of culture supernatant was collected at indicated timepoints. Serial 10-fold dilutions were made in duplicate and applied to BHK-21 cell monolayers. Virus adsorption was allowed for 1 h at 37 °C, following which the inoculum was removed, and the cell monolayers were overlaid with MEM containing 1% low-gelling-temperature (LGT) agarose, 2% FBS and 1% antibiotic solution. After incubation for 16 h at 37 °C and 5% CO_2_, cells were fixed and the virus was inactivated in 10% formaldehyde in PBS. The agarose plugs were removed, and the monolayers were stained with 0.1% crystal violet in 30% methanol. Plaques were counted manually.

### 2.2. Antibodies and Other Reagents

For the detection of viral proteins, mouse anti-VSV antibody was used as previously described [20]. Anti-G6PD (cat. no. ab129199) was obtained from Abcam (Cambridge Biomedical Campus, Cambridge, UK). Anti-β-actin (cat. no. A2228), horseradish peroxidase (HRP) conjugated goat anti-rabbit (cat. no. A6154), and goat anti-mouse (cat. no. A4416) secondary antibodies and propidium iodide (PI) were purchased from Sigma-Aldrich (St. Loius, MO, USA). FK866, nicotinamide, nicotinic acid, L-tryptophan, 2-DG, and D-ribose were obtained from Thermo Scientific (Waltham, MA, USA). Nicotinamide riboside was purchased from Combi-Blocks (San Diego, CA, USA). 6-AN was obtained from Alfa Aesar (Haverhill, MA, USA), and BPTES was obtained from Cayman Chemical (Ann Arbor, MI, USA).

Unless otherwise specified, the final concentrations of drugs and inhibitors used were as follows: 2-DG, 5, 25, and 50 mM; 6-AN, 0.25, 0.5, and 1 mM; BPTES, 1, 5, and 10 µM; D-ribose, 25 mM; nicotinamide riboside, 300 µM; L-tryptophan, 300 µM; nicotinic acid, 300 µm; and nicotinamide, 300 µM. 2-DG, D-ribose, nicotinamide riboside, tryptophan, nicotinic acid, and nicotinamide were dissolved in water, while 6-AN and BPTES were dissolved in dimethyl sulfoxide (DMSO).

### 2.3. Virus Infection and Drug Treatment

For pharmacological inhibition of glycolysis, PPP or glutaminolysis, cells were left untreated or pretreated with indicated concentrations of 2-DG, 6-AN or BPTES respectively for 1 h. Cells were then infected with VSV-PeGFP at an MOI of 0.01 for 1 h to allow viral adsorption. Following infection, cells were washed with PBS and overlaid with 1 mL of DMEM supplemented with 2% FBS and the corresponding concentrations of the inhibitors and incubated for further 24 h before harvesting.

### 2.4. Flow Cytometry

Cell viability was assessed by simultaneous determination of PI staining and cell size (forward scatter properties). Data collection for cell viability and percentage of GFP- positive cells was done using a CytoFLEX flow cytometer (Beckman Couter, Brea, CA, USA) and data were analyzed using CytExpert software, version 2.6.

### 2.5. siRNA-Mediated Protein Depletion

Knockdown of G6PD expression was achieved using a pool of small interfering RNAs (siRNAs) targeting G6PD (cat. no. JL-008181-02-0005; Horizon Discovery, Waterbeach, UK). A final concentration of 80 nM siRNA was transfected in cells using Lipofectamine™ RNAiMAX (Invitrogen, Carlsbad, CA, USA) according to the manufacturer’s protocol. A non-targeting (NT) siRNA (cat. no. 1027281; Qiagen, Hilden, Germany) served as a negative control. At 24 h post-transfection, the culture medium was replaced with DMEM supplemented with 2% FBS and 1× antibiotic solution, and the cells were further incubated for 24 h before VSV-PeGFP infection.

### 2.6. Cell Lysate Preparation and Immunoblot Analysis

Cells were harvested at 24 h post-infection (hpi) and lysed in 1× radioimmunoprecipitation assay (RIPA) buffer (25 mM Tris-HCl, pH 7.6; 150 mM NaCl; 1% Triton X-100; 1% sodium deoxycholate; 0.1% SDS) supplemented with the Halt™ protease and phosphatase inhibitor cocktail (cat no. 1861281; Thermo Scientific). The lysates were incubated on ice for 5 min and clarified at 12,000× *g* for 10 min. Total protein was quantified by a bicinchoninic acid (BCA) assay kit (Thermo Scientific, Waltham, MA, USA). Thirty micrograms of total protein was separated by sodium dodecyl sulphate–polyacrylamide gel electrophoresis (SDS–PAGE) and transferred onto polyvinylidene difluoride (PVDF) membrane. Non-specific protein binding was blocked for 1 h with 5% (*w*/*v*) skim milk prepared in Tris-buffered saline containing 0.2% Tween-20 (TBST) and incubated overnight at 4 °C with primary antibodies against VSV, G6PD, or β-actin. The membrane was washed with TBST and incubated for 2 h at room temperature with appropriate HRP-conjugated secondary antibodies. Membrane was washed with TBST, and protein bands were visualized using enhanced chemiluminescence (ECL) Western blotting substrate (cat no. 32106; Thermo Scientific). Images were taken with a ChemiDoc™ imaging system (Bio-Rad, Hercules, CA, USA).

### 2.7. Statistical Analysis

All experiments were performed as independent replicates. Data were analyzed using one-way or two-way analysis of variance (ANOVA) with GraphPad Prism software (ver. 10). When the assumptions of ANOVA were not met (with normality assessed by the Shapiro–Wilk test or equal variance), either one-way ANOVA on ranks or appropriate data transformations (for two-way ANOVA) were applied. Results are presented as the mean ± standard deviation (SD).

## 3. Results

### 3.1. Inhibition of Glycolysis Suppresses VSV Replication

Early investigations established a link between host glucose metabolism and VSV replication. Pharmacological inhibition of glycolysis using 2-DG, a glucose analog that interferes with hexokinase activity and glycolytic flux, was shown to markedly suppress VSV replication, implicating glycolysis as an important determinant of viral growth [16,17]. While these foundational studies clearly demonstrated the antiviral effect of glycolytic inhibition, they did not provide mechanistic insight into how disruption of glycolysis impairs VSV replication. Moreover, the extent to which glycolysis supports VSV replication in metabolically reprogrammed cancer cells—such as glioblastoma cells that rely heavily on aerobic glycolysis—remains poorly defined. Although recent work has shown that the VSV matrix (M) protein can promote glycolytic metabolism in cancer cells [14], the direct consequences of glycolytic inhibition on VSV replication in this context have not been systematically examined.

**Figure 1:**
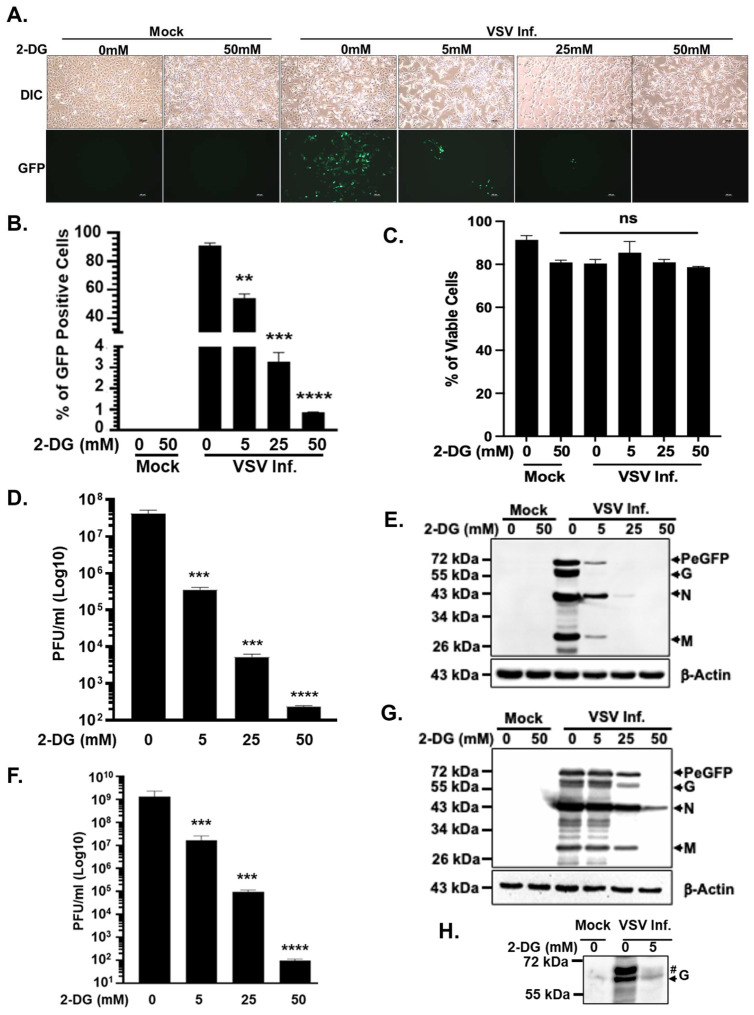
Inhibition of glycolysis with 2-DG blocks VSV replication. (**A**) A172 cells were mock-infected or infected with VSV-PeGFP at an MOI of 0.01. Representative fluorescence microscopy images depicting GFP expression in untreated cells or in cells treated with varying concentrations of 2-DG were collected at 24 hpi. (**B**) The proportion of GFP-positive A172 cells was quantified by flow cytometry at 24 hpi. (**C**) Viability of A172 cells following 2-DG treatment and VSV infection was assessed at 24 hpi. (**D**) Viral titers in culture supernatants after 2-DG treatment, measured by plaque assay in A172 cells. (**E**) Western blot analysis of VSV proteins in A172 cells, with β-actin levels revealing loading control. (**F**) Viral titers in culture supernatants after 2-DG treatment in BHK-21 cells, measured by plaque assay. (**G**) Western blot analysis of VSV proteins, with β-actin shown as a loading control in BHK-21 cells. (**H**) Western blot analysis of VSV G protein glycosylation status in BHK-21 cells untreated or treated with indicated concentrations of 2-DG. Fully glycosylated (indicated by ‘#’) and unglycosylated (indicated by the arrow) bands are shown. Data in the bar plots represent the mean ± SD from three independent biological replicates. Western blots are from representative experiments. Statistical significance was determined using ordinary one-way ANOVA, with comparisons made relative to the mock-infected or untreated control group. Significance is indicated as ** (*p* < 0.01), *** (*p* < 0.001), and **** (*p* < 0.0001); non-significant, ns (*p* > 0.05).

To evaluate the requirement for glycolysis during VSV infection in glioblastoma cells, we treated A172 cells with increasing concentrations of 2-DG and assessed viral replication following infection with an enhanced green fluorescent protein-tagged VSV (VSV-PeGFP), in which eGFP is fused in-frame to the viral phosphoprotein (P) [18]. Inhibition of glycolysis by 2-DG resulted in a pronounced and dose-dependent reduction in VSV replication. Fluorescence microscopy revealed a substantial decrease in GFP signal in infected cells treated with increasing doses of 2-DG compared to untreated controls, indicating a strong suppression of viral gene expression (Figure 1A). Quantitative analysis further demonstrated a significant reduction in the percentage of eGFP-positive cells as 2-DG concentrations increased (Figure 1B).

Because inhibition of glycolytic flux by 2-DG can induce cellular stress responses and, at higher concentrations or prolonged exposure, lead to cytotoxicity and cell death [21], we next assessed whether the observed antiviral effects were secondary to compromised cell viability. Flow cytometric analysis of cell viability revealed that 2-DG treatment at concentrations sufficient to suppress viral replication did not significantly reduce cell viability over the course of infection (Figure 1C). These findings indicate that the reduction in viral gene expression was not attributable to generalized cytotoxic effects of 2-DG.

Consistent with the observed inhibition of viral gene expression, analysis of infectious progeny production demonstrated a robust, dose-dependent decrease in virus yield. Treatment with 5, 25, or 50 mM 2-DG resulted in approximately 2, 4, or 5 Log_10_ reductions in viral titers, respectively (Figure 1D). Immunoblot analysis further confirmed a marked reduction in the accumulation of VSV structural proteins in infected cells treated with increasing concentrations of 2-DG (Figure 1E). Importantly, the inhibitory effect of glycolytic blockade was not restricted to glioblastoma cells. Similar reductions in infectious virus production (Figure 1F) and viral protein expression (Figure 1G) were observed in BHK-21 cells, a cell line commonly used for VSV propagation, indicating that the requirement for glycolysis is not cell-type-specific. Furthermore, because 2-DG disrupts both glycolytic flux and N-linked glycosylation [22,23], we assessed whether impaired glycoprotein processing might have contributed to the observed reduction in infectious virus production. Immunoblot analysis of VSV-G in BHK-21 cells demonstrated altered electrophoretic mobility under 2-DG treatment, consistent with impairment in glycosylation (Figure 1H). In A172 cells, however, 2-DG treatment markedly reduced overall viral protein levels, precluding reliable assessment of G protein glycosylation status. Thus, while impaired glycoprotein maturation may contribute to reduced viral yield, the global suppression of viral protein expression suggests that metabolic stress broadly limits VSV replication under these conditions.

Collectively, these results demonstrate that inhibition of glycolysis selectively impairs VSV replication independently of overt cytotoxicity. Together with earlier observations and recent findings linking VSV infection or the viral M protein to metabolic reprogramming [14,16,17], these data establish glycolysis as a critical host metabolic pathway required for efficient VSV replication in glioblastoma cells.

### 3.2. Requirement of Pentose Phosphate Pathway for VSV Replication Is Less Stringent than That of Glycolysis

Because glycolysis is metabolically coupled to the pentose phosphate pathway (PPP), supplying precursors for nucleotide biosynthesis and maintaining cellular redox balance through NADPH production, we next examined whether selective disruption of the PPP similarly impairs VSV replication. To interrogate the contribution of this interconnected metabolic node, we treated cells with 6-AN, a well-characterized inhibitor of glucose-6-phosphate dehydrogenase-dependent PPP activity and subsequently assessed virus replication following VSV infection.

**Figure 2:**
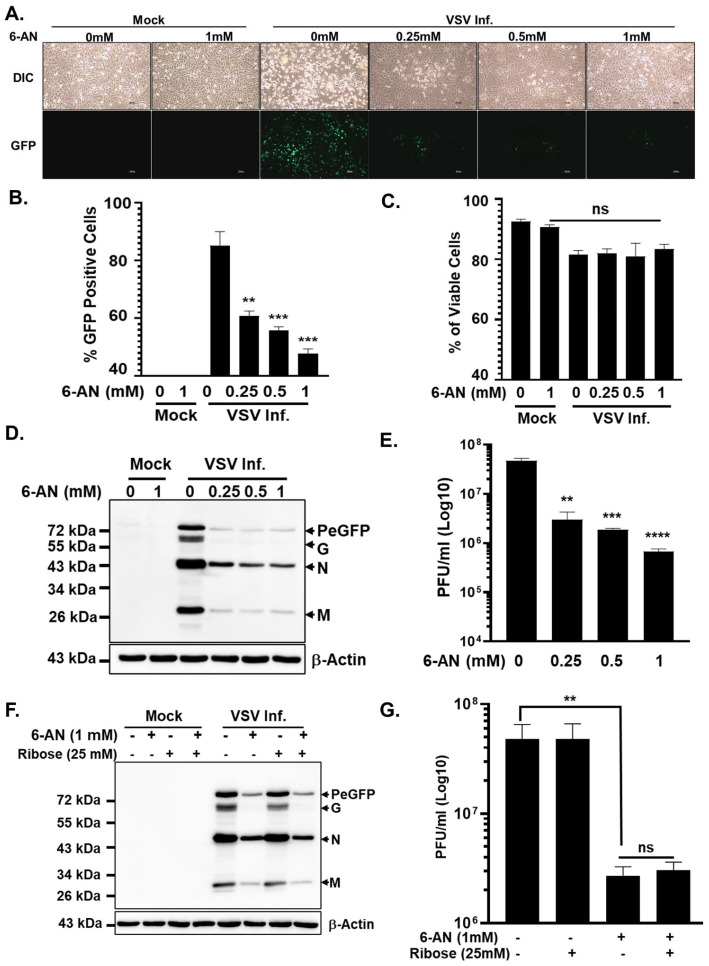
Perturbation of PPP negatively impacts VSV replication. (**A**) Representative fluorescence microscopy images showing GFP expression in untreated and 6-AN-treated cells infected with VSV-PeGFP as described in Figure 1. (**B**) Quantification of the percentage of GFP-positive cells following VSV-PeGFP infection. (**C**) Percentage of viable cells following 6-AN treatment and VSV-PeGFP infection. (**D**) Western blot analysis of VSV proteins, with β-actin levels showing loading control. (**E**) Viral titers in the culture supernatants after 6-AN treatment, measured by plaque assay. (**F**) Western blot analysis of VSV protein expression following 6-AN treatment with or without D-ribose supplementation. (**G**) Viral titers in the culture supernatant under the indicated treatment conditions, quantified by plaque assay. Data in the bar plots represent the mean ± SD from three independent biological replicates. Western blots are from representative experiments. Statistical significance was determined using ordinary one-way ANOVA, with comparisons made relative to the mock-infected or untreated control group. ‘-’ and ‘+’ refer to ‘untreated’ and ‘treated’ with the corresponding compounds. Significance is indicated as ** (*p* < 0.01), *** (*p* < 0.001), and **** (*p* < 0.0001); non-significant, ns (*p* > 0.05).

Treatment of A172 cells with 6-AN resulted in a significant and reproducible inhibition of VSV replication, albeit to a lesser extent than that observed with glycolytic blockade. Fluorescence microscopy demonstrated a clear reduction in GFP expression in VSV-infected cells exposed to 6-AN compared to untreated controls, indicating impaired viral gene expression (Figure 2A). Consistent with these observations, flow cytometric analysis revealed a decrease in the percentage of GFP-positive cells following 6-AN treatment (Figure 2B). Importantly, these effects were observed at drug concentrations that did not significantly compromise cell viability, as confirmed by viability analysis (Figure 2C), indicating that the reduction in viral replication was not attributable to nonspecific cytotoxicity.

Immunoblot analysis further demonstrated a dose-dependent decrease in the accumulation of VSV proteins in infected cells treated with 6-AN (Figure 2D). In parallel, measurement of infectious progeny production revealed a significant reduction in viral titers in the supernatants of 6-AN-treated cultures (Figure 2E). Notably, despite these inhibitory effects, VSV gene expression and infectious virus production were not completely abrogated, indicating that PPP inhibition imposes a partial constraint on viral replication. In contrast to the profound suppression observed upon glycolytic inhibition with 2-DG, these results suggest that the requirement for PPP activity during VSV replication is comparatively less stringent.

Given the central role of the PPP in ribose-5-phosphate generation and de novo nucleotide biosynthesis, we next sought to determine whether reduced nucleotide availability underlies the inhibitory effects of 6-AN on VSV replication. To address this possibility, we performed rescue experiments using exogenous D-ribose supplementation in 6-AN-treated cells. D-ribose can bypass the oxidative branch of the PPP by replenishing ribose-5-phosphate pools downstream of glucose-6-phosphate dehydrogenase inhibition, thereby supporting nucleotide biosynthesis and cellular energy metabolism [24,25].

However, supplementation with D-ribose failed to restore VSV replication in 6-AN-treated cells, as assessed by both viral protein accumulation and infectious virus production (Figure 2F,G). These findings indicate that impaired ribose availability and nucleotide biosynthesis are unlikely to be the primary mechanisms responsible for the suppressive effects of PPP inhibition on VSV replication. Instead, these results suggest that other PPP-dependent functions, such as NADPH production and redox homeostasis, may contribute to optimal VSV replication, although these requirements appear less critical than the glycolytic flux itself.

Collectively, these data demonstrate that while the PPP supports efficient VSV replication, its contribution is subordinate to that of glycolysis, highlighting a differential dependence of VSV on host metabolic pathways.

### 3.3. Depletion of G6PD Enhances VSV Growth

The inability of exogenous D-ribose to rescue the inhibitory effects of 6-AN on VSV replication suggests that cellular processes beyond impaired ribose-5-phosphate production and nucleotide biosynthesis contribute to the observed antiviral phenotype. Because 6-AN is known to exert pleiotropic effects on cellular metabolism and redox homeostasis, including interference with NADP^+^/NADPH-dependent enzymes and induction of oxidative stress [26], we sought to more specifically interrogate the role of the PPP in VSV replication using a genetic approach.

**Figure 3:**
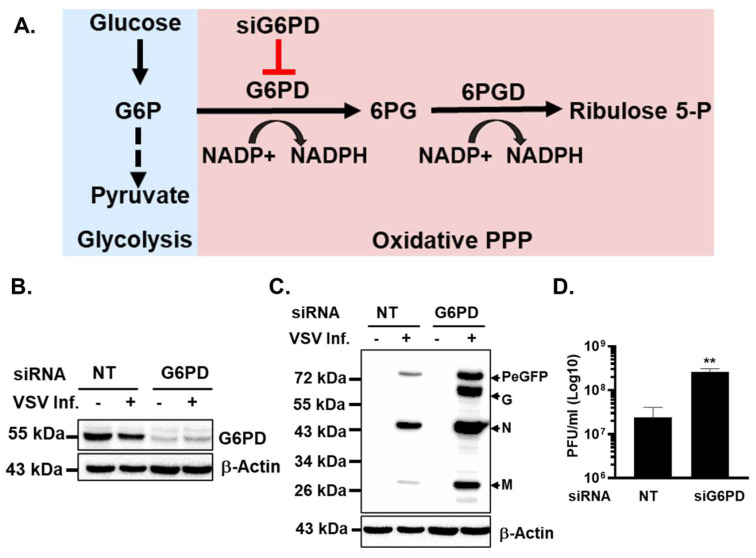
Depletion of G6PD enhances VSV replication in A172 cells. (**A**) Schematic of oxidative PPP showing G6PD as the key enzyme in the pathway. Red T-bar refers to ‘blockage/inhibition’; discountinious black arrow refers to ‘multiple steps’ in the process and curved black arrow refers to ‘conversion’. (**B**) Western blot analysis of G6PD protein accumulation in cells transfected with NT siRNA or G6PD-targeting siRNA. (**C**) Western blot detection of VSV proteins in uninfected (-) or virus-infected (+) cells under the indicated siRNA treatment conditions. (**D**) Infectious VSV titers in culture supernatants of siRNA-treated and VSV-PeGFP-infected cells, quantified by plaque assay. The data in the plot represents the mean ± SD from three independent biological replicates. Western blots are from representative experiments. Statistical significance was determined using ordinary one-way ANOVA, with comparisons made relative to the NT or G6PD siRNA group. Statistical significance is indicated as ** (*p* < 0.01).

To this end, we selectively depleted glucose-6-phosphate dehydrogenase (G6PD), the rate-limiting enzyme of the oxidative branch of the PPP, using small interfering RNA (siRNA) in A172 cells. G6PD catalyzes the conversion of glucose-6-phosphate to 6-phosphogluconolactone, concomitantly generating NADPH, which is essential for maintaining cellular redox balance and supporting biosynthetic reactions [27] (Figure 3A). Immunoblot analysis confirmed efficient and specific depletion of G6PD protein in cells treated with G6PD-targeting siRNA compared to non-targeting (NT) control siRNA (Figure 3B).

Unexpectedly, and in stark contrast to the effects observed with pharmacological PPP inhibition, siRNA-mediated depletion of G6PD resulted in a significant enhancement of VSV replication. G6PD-depleted cells exhibited markedly increased viral protein expression relative to NT siRNA-treated controls, as assessed by immunoblotting (Figure 3C). This increase in viral protein accumulation was accompanied by a corresponding elevation in the production of infectious progeny virus, as determined by viral titration assays (Figure 3D).

Taken together, these findings reveal a striking dichotomy between pharmacological inhibition of the PPP and genetic depletion of G6PD with respect to VSV replication. While 6-AN treatment suppresses VSV gene expression and virus production, specific depletion of G6PD enhances viral replication, indicating that reduced PPP flux per se does not limit VSV growth. Instead, these data suggest that the antiviral effects of 6-AN are likely mediated through additional cellular mechanisms independent of direct PPP inhibition. Such mechanisms may include altered redox signaling, induction of stress responses, or disruption of NADPH-dependent antiviral pathways, which have been shown to influence RNA virus replication and innate immune responses [28,29].

Collectively, these results underscore the importance of distinguishing between pathway-specific metabolic requirements and off-target or pleiotropic effects of metabolic inhibitors, and they identify G6PD activity as a potential host restriction factor whose depletion paradoxically enhances VSV replication.

### 3.4. 6-AN Depletes Cellular NAD^+^ Pool and Inhibits VSV Replication

The opposing effects of pharmacological inhibition of the PPP by 6-AN and siRNA-mediated depletion of the PPP enzyme G6PD prompted us to further investigate the mechanism underlying the antiviral activity of 6-AN. In addition to its role in nucleotide biosynthesis and redox balance, the PPP is functionally linked to nicotinamide adenine dinucleotide (NAD^+^) metabolism, which is essential for cellular energy homeostasis, redox reactions, and multiple signaling pathways that influence virus replication [30,31]. These observations raise the possibility that 6-AN suppresses VSV replication through disruption of intracellular NAD^+^ homeostasis rather than through direct inhibition of PPP-derived biosynthetic outputs.

**Figure 4:**
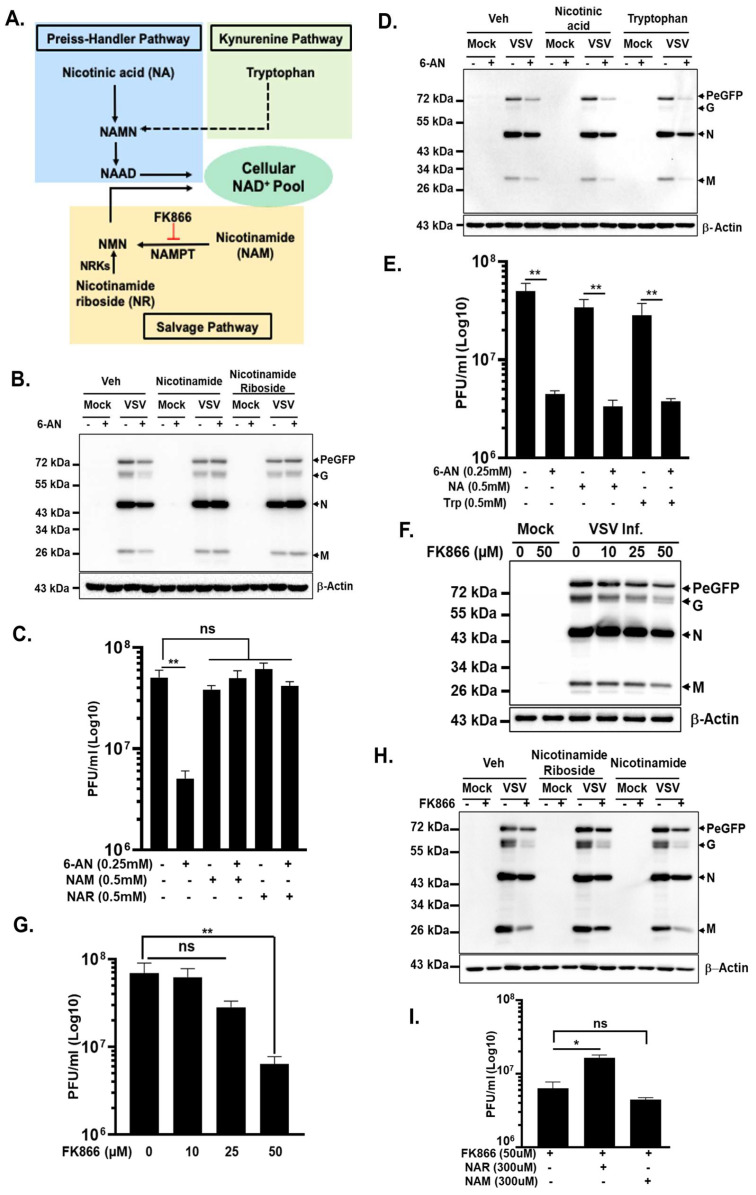
Effects of NAD^+^ precursors on 6-AN-mediated inhibition of VSV replication. (**A**) Schematic showing the different pathways leading to the synthesis and/or recycling of NAD^+^. Generation of NAD^+^ through various pathways from tryptophan, nicotinic acid, nicotinamide, and nicotinamide riboside as shown. Tryptophan feeds into NAD^+^ synthesis via the de novo pathway, whereas nicotinic acid is utilized through the Preiss–Handler pathway. The salvage pathway converts nicotinamide to nicotinamide mononucleotide (NMN) via the rate-limiting enzyme NAMPT, which is inhibited by FK866. Nicotinamide riboside is converted to NMN by nicotinamide riboside kinases (NRKs), bypassing NAMPT. Red T-bar refers to ‘blockage/inhibition’; discountinious black arrow refers to ‘multiple steps’ in the process. (**B**) Western blot analysis of viral protein expression in VSV-PeGFP-infected cells after 6-AN treatment in the presence of nicotinamide or nicotinamide riboside. (**C**) Corresponding viral titers in the culture supernatant following treatment with 6-AN and the indicated precursors, determined by plaque assay. (**D**) Western blot analysis of viral protein expression in VSV-PeGFP-infected cells after 6-AN treatment in the presence of nicotinic acid or tryptophan. (**E**) Produced progeny titers in the culture supernatant following treatment with 6-AN and the indicated precursors, determined by plaque assay. (**F**) Western blot analysis of viral protein expression in VSV-PeGFP-infected cells following treatment with FK866. (**G**) Infectious VSV titers in culture supernatants after FK866 treatment, quantified by plaque assay. (**H**) Western blot analysis of viral proteins after FK866 treatment in the presence of the NAD^+^ salvage pathway precursors nicotinamide riboside and nicotinamide. (**I**) Infectious VSV titers under indicated conditions quantified by plaque assay. Veh, vehicle. ‘-’ and ‘+’ refer to ‘untreated’ and ‘treated’ with the corresponding compounds. Data in the bar plots represent the mean ± SD from three independent biological replicates. Western blots are from representative experiments. Statistical significance was determined using ordinary one-way ANOVA, with comparisons made relative to the mock-infected or untreated control group. Significance is indicated as * (*p* < 0.05) and ** (*p* < 0.01); non-significant, ns (*p* > 0.05).

NAD^+^ can be synthesized from four major precursors in mammalian cells: nicotinamide (NAM), nicotinamide riboside (NR), tryptophan, and nicotinic acid (Figure 4A). De novo NAD^+^ synthesis from tryptophan proceeds through the kynurenine pathway and requires multiple enzymatic steps, while nicotinic acid contributes to NAD^+^ production via the Preiss–Handler pathway. However, in most mammalian cells, the predominant source of NAD^+^ is the salvage pathway, which regenerates NAD^+^ from NAM, a by-product of NAD^+^-consuming reactions, or from exogenous NR [30,31]. Importantly, 6-AN is a structural analog and anti-metabolite of NAM, and our recent metabolomic analyses have demonstrated that 6-AN broadly depletes intracellular NAD^+^ pools [28].

Based on these observations, we hypothesized that the antiviral effect of 6-AN is driven, at least in part, by disruption of NAD(H) metabolism. To test this, A172 cells were infected with VSV in the presence of 6-AN and supplemented with either NAM or NR, two precursors that replenish the intracellular NAD^+^ pool through the salvage pathway (Figure 4A). Immunoblot analysis revealed that supplementation with either NAM or NR substantially restored VSV protein accumulation in 6-AN-treated cells compared to cells treated with 6-AN alone (Figure 4B). Consistent with the restoration of viral protein expression, infectious virus production was also significantly rescued in the presence of either NAM or NR (Figure 4C).

To further dissect the pathway specificity of this rescue, we next evaluated NAD^+^ precursors that feed into distinct biosynthetic routes. Supplementation with tryptophan, which supports de novo NAD^+^ synthesis via the kynurenine pathway, or with nicotinic acid, which enters the Preiss–Handler pathway, failed to restore VSV replication in 6-AN-treated cells (Figure 4D,E). These results suggest that the suppressive effects of 6-AN on VSV replication are specifically linked to disruption of NAD^+^ salvage pathway-mediated NAD^+^ replenishment rather than global impairment of all NAD^+^ biosynthetic routes.

Given that the salvage pathway is the primary source of NAD^+^ in mammalian cells, we next examined whether direct depletion of intracellular NAD^+^ is sufficient to impair VSV replication. To this end, cells were treated with FK866, a selective and potent inhibitor of nicotinamide phosphoribosyltransferase (NAMPT), the rate-limiting enzyme in the NAD^+^ salvage pathway that converts NAM to nicotinamide mononucleotide (NMN) (Figure 4A) [32,33]. Immunoblot analysis revealed a dose-dependent reduction in VSV protein accumulation following FK866 treatment, with pronounced inhibition observed at 50 μM FK866 (Figure 4F). In parallel, infectious virus production was reduced in a dose-dependent manner, mirroring the decrease in viral protein expression (Figure 4G). Although modest reductions were reproducibly observed at lower concentrations of FK866, substantial inhibition of VSV replication required higher drug concentrations.

Because NAD^+^ synthesis in mammalian cells is largely dependent on NAM and NR, we next tested whether supplementation with these precursors could rescue VSV replication under conditions of FK866-mediated inhibition. Consistent with the mechanism of action of FK866, supplementation with NAM failed to restore viral protein accumulation and infectious virus yield, as NAMPT activity is required to convert NAM into NMN (Figure 4H,I). In contrast, NR is phosphorylated to NMN by nicotinamide riboside kinases (NRKs), thereby bypassing NAMPT and feeding directly into NAD^+^ synthesis (Figure 4A). Accordingly, supplementation with NR partially restored viral protein expression and infectious virus titers in FK866-treated cells (Figure 4H,I), as judged by the increased detection of the viral proteins in the presence of FK866 and NR compared to that with FK866 alone.

Collectively, these findings demonstrate that intracellular NAD^+^ availability is a critical determinant of efficient VSV replication. Pharmacological depletion of NAD^+^ pools through 6-AN or NAMPT inhibition suppresses viral gene expression and infectious virus production, whereas selective restoration of NAD^+^ levels through salvage pathway precursors rescues viral growth. These data identify NAD^+^ metabolism as a key host metabolic vulnerability exploited by VSV and provide a mechanistic explanation for the antiviral activity of 6-AN independent of direct PPP inhibition.

### 3.5. Glutaminolysis Is Required for Efficient VSV Replication

Glutamine metabolism is a major contributor to central carbon metabolism in cancer cells, where it serves as a critical anaplerotic substrate for the tricarboxylic acid (TCA) cycle and supports ATP generation, macromolecular biosynthesis, and redox homeostasis. In many tumors, including glioblastoma, glutamine-derived carbon and nitrogen are essential for sustaining mitochondrial metabolism, maintaining pools of TCA cycle intermediates, and generating reducing equivalents such as NADH and NADPH [34,35,36]. Glioblastoma cells are particularly dependent on glutamine to fuel oxidative metabolism and compensate for truncated glucose oxidation, a metabolic state that has been described as “glutamine addiction” [37,38]. Given this pronounced reliance on glutamine-driven anaplerosis, we next examined whether efficient VSV replication requires glutaminolysis in glioblastoma cells.

To selectively inhibit glutamine utilization, cells were treated with BPTES, a well-characterized allosteric inhibitor of glutaminase (GLS), the mitochondrial enzyme that catalyzes the conversion of glutamine to glutamate, the first committed step of glutaminolysis [39,40]. A172 cells were subsequently infected with VSV in the continuous presence of BPTES, and viral replication was assessed using fluorescence microscopy, immunoblot analysis, and plaque assays.

**Figure 5:**
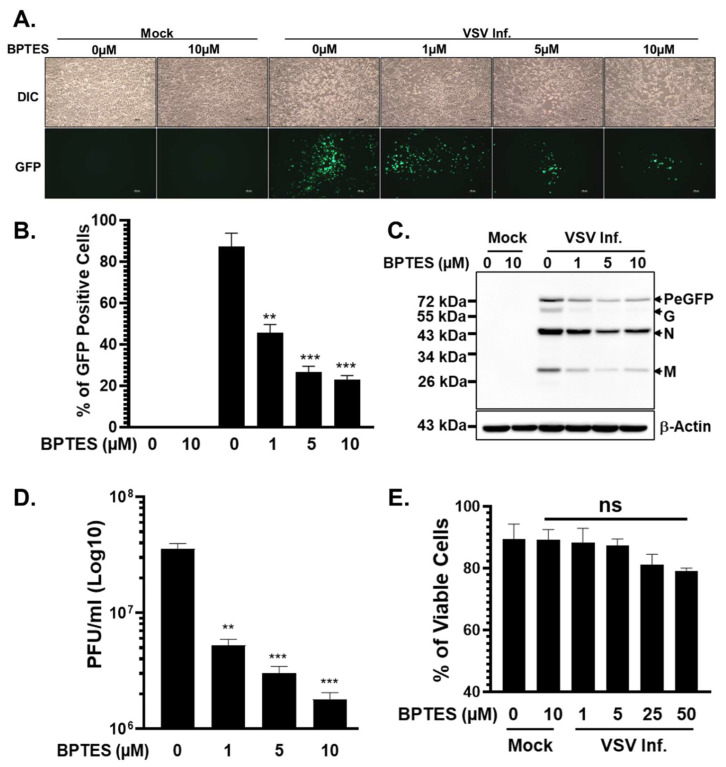
Inhibition of glutaminolysis by BPTES suppresses VSV replication. (**A**) Representative fluorescence microscopy images of VSV-PeGFP-infected cells showing GFP expression in control and BPTES-treated cells. (**B**) Quantification of the percentage of GFP-positive cells in BPTES-treated and infected cells. (**C**) Western blot analysis of viral proteins, with β-actin levels showing loading control. (**D**) Infectious viral titers in culture supernatants after BPTES treatment, determined by plaque assay. (**E**) Cell viability following BPTES treatment during VSV infection. Data in bar plots represent the mean ± SD from three independent biological replicates. Statistical significance was determined using ordinary one-way ANOVA, with comparisons made relative to the mock-infected or untreated control group. Significance is indicated as ** (*p* < 0.01) and *** (*p* < 0.001). Non-significant, ns (*p* > 0.05).

Inhibition of glutaminolysis by BPTES resulted in a pronounced, dose-dependent reduction in VSV replication. Fluorescence microscopy revealed markedly diminished GFP expression and a significant decrease in the number of GFP-positive cells in BPTES-treated cultures compared to vehicle-treated controls (Figure 5A,B), indicating strong suppression of viral gene expression. Consistent with these observations, immunoblot analysis demonstrated reduced accumulation of VSV proteins in infected cells exposed to BPTES (Figure 5C). Quantification of infectious progeny further confirmed that disruption of glutamine metabolism significantly impaired productive virus replication, as evidenced by substantially reduced VSV titers in the supernatants of BPTES-treated cells (Figure 5D).

Importantly, BPTES treatment did not adversely affect cell viability at the concentrations used during the course of infection (Figure 5E), indicating that the observed antiviral effects were not due to nonspecific cytotoxicity. Rather, these findings suggest that glutamine-derived carbon flux directly supports VSV replication, likely by sustaining mitochondrial metabolism, energy production, and biosynthetic capacity required for viral gene expression and assembly.

Together, these data demonstrate that glutaminolysis is a critical host metabolic pathway supporting efficient VSV replication in glioblastoma cells. In the context of the well-established glutamine dependence of glioblastoma [37,38], these findings highlight how tumor-specific metabolic states can shape oncolytic virus biology. Disruption of glutamine-driven central carbon metabolism suppresses VSV replication, revealing a metabolic vulnerability that may have important implications for the rational combination of glutamine-targeted therapies with oncolytic VSV-based treatment strategies.

## 4. Discussion

**Figure 6:**
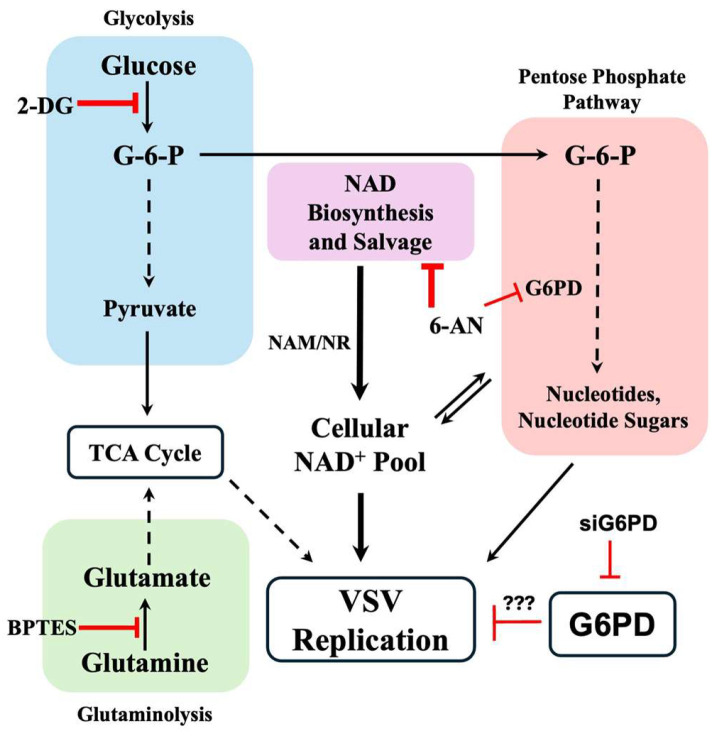
Summary of central carbon metabolic pathways impacting VSV replication. The dashed arrows indicate multiple steps in the respective pathways. Red T-bars refer to ‘blockage/inhibition’ and ‘???’ refers to unknown mechanism.

Viruses extensively reprogram host cell metabolism to generate energy, reducing equivalents, and biosynthetic precursors required for genome replication, transcription, protein synthesis, and virion assembly [41,42]. In cancer cells, these metabolic pathways are already profoundly rewired to support uncontrolled proliferation and survival, creating a metabolic environment that can strongly influence virus–host interactions and oncolytic virus performance [15,43,44,45]. In this study, we demonstrate that VSV replication is highly sensitive to perturbations in central carbon metabolism, including glycolysis, the PPP, and glutaminolysis (Figure 6). These findings are consistent with a growing body of literature showing that many RNA and DNA viruses depend on specific metabolic pathways to establish productive infection and spread [42,43,44,46,47]. Importantly, our observation that intracellular NAD^+^ availability is essential for efficient VSV replication highlights a redox-centric framework in which metabolic homeostasis, rather than simple substrate availability, is a key determinant of productive infection. Together, our data indicate that VSV replication is exquisitely sensitive to glycolytic flux and intracellular NAD^+^ pools, underscoring the central role of metabolic state in shaping virus replication outcomes.

Among the metabolic interventions tested, inhibition of glycolysis by 2-DG produced the most profound suppression of VSV replication. Although 2-DG is widely used as a glycolytic inhibitor, it also interferes with N-linked glycosylation by disrupting the synthesis of lipid-linked oligosaccharide precursors in the endoplasmic reticulum [22,23]. This dual activity has important implications for enveloped viruses such as VSV, whose infectivity critically depends on proper glycosylation of the viral glycoprotein G. Our observations that viral protein synthesis and infectious progeny production are profoundly reduced in the presence of 2-DG (Figure 1), together with the nearly undetectable levels of fully glycosylated G protein under these conditions, suggest that impaired glycoprotein maturation contributes substantially to the antiviral effect. Similar inhibition of glycosylation and progeny virus production has also been observed for viruses such as LCMV, SARS-CoV-2 and other pathogenic viruses [46,47,48]. These findings confirm and extend early studies demonstrating reduced VSV progeny production following 2-DG treatment [16,17] and reinforce the concept that targeting upstream metabolic pathways such as glycolysis can exert strong antiviral effects through combined disruption of energy production and post-translational processing.

Many viruses actively induce aerobic glycolysis—often described as a virus-induced Warburg effect—to meet increased energetic and biosynthetic demands during infection [43,44]. Metabolomic and flux analyses have shown that infection by diverse viruses, including dengue virus, herpesviruses, and coronaviruses, is associated with increased glycolytic flux and diversion of glycolytic intermediates into biosynthetic pathways [41,42,46,49,50,51]. Dengue virus, for example, promotes glycolysis and redirects carbon into the PPP to support nucleotide biosynthesis and NADPH generation required for genome replication and mitigation of oxidative stress [49]. Similarly, SARS-CoV-2 infection enhances glycolytic and PPP activity, and inhibition of these pathways suppresses viral replication [51,52]. Our data suggest that VSV likewise relies heavily on glycolysis but may be uniquely constrained by glycosylation requirements, highlighting a vulnerability specific to enveloped viruses whose entry and egress depend on properly processed viral glycoproteins. In contrast, inhibition of the PPP or glutaminolysis produces more modest, though still significant, reductions in viral replication, indicating that these pathways play supportive rather than strictly essential roles during VSV infection.

The PPP provides ribose-5-phosphate for nucleotide biosynthesis and NADPH for reductive biosynthesis and antioxidant defense. Interestingly, supplementation with D-ribose failed to rescue VSV replication in 6-AN-treated cells, arguing against nucleotide depletion as the primary mechanism underlying the antiviral effect of 6-AN. Instead, our data point to disruption of NAD(P)(H) homeostasis as a key determinant. Many viruses upregulate PPP flux to meet demands for NADPH and to counteract oxidative stress generated during infection [43,53]. However, our findings indicate that broad pharmacological inhibition of the PPP is less detrimental to VSV replication than disruption of glycolysis and glycosylation, suggesting that VSV relies more heavily on glycolysis-derived carbon flux and post-translational processing than on de novo nucleotide synthesis per se.

A particularly intriguing finding from this study is that genetic depletion of G6PD enhanced VSV replication, revealing a complex and context-dependent role for the PPP in host–virus interactions. This seemingly paradoxical effect may reflect alterations in cellular redox balance and innate immune signaling. G6PD deficiency reduces NADPH production and can increase oxidative stress, which in some contexts suppresses antiviral responses, including interferon signaling and ROS-dependent antiviral pathways [29,54]. Thus, partial or transient suppression of PPP activity may inadvertently weaken antiviral defenses, thereby facilitating VSV replication. In this context, G6PD may function as a host restriction factor that limits VSV replication under basal conditions. However, the precise mechanism by which G6PD constrains viral replication remains unresolved, and further studies will be required to determine whether this effect is mediated through redox regulation, modulation of innate immune signaling, or other PPP-dependent processes. Similar complexities have been observed in other viral systems. For example, inhibition of the PPP with 6-AN suppresses Zika virus replication not primarily by limiting ribose production, but by depleting NAD(H) pools and impairing NAD-dependent metabolic reactions—effects that are reversible by NAD^+^ salvage pathway precursors [28]. Our findings with VSV align with this redox-centric view observed for other viruses [55,56] and underscore the importance of NAD^+^/NADH balance, rather than simple metabolic flux, in supporting productive infection.

Viral infection frequently perturbs NAD^+^ homeostasis through increased metabolic demand and activation of NAD^+^-consuming enzymes such as PARPs and sirtuins, which are integral components of antiviral innate immune responses [42,55,57,58]. Maintenance of NAD^+^ levels may therefore play a dual role: supporting metabolic processes essential for viral propagation while simultaneously sustaining host antiviral defenses. The observed rescue of VSV replication by nicotinamide riboside (NR) in FK866-treated cells indicates that replenishing NAD^+^ pools can overcome metabolic bottlenecks imposed by NAMPT inhibition, likely by restoring oxidoreductase activity, ATP production, and redox balance required for viral RNA synthesis and protein translation. Consistent with this interpretation, inhibition of NAD^+^ biosynthesis via FK866 suppressed VSV replication, while supplementation with NR—which bypasses the NAMPT step—partially restored viral growth. Similar dependencies have been reported for other viruses; dengue virus replication is impaired by NAD^+^ depletion and rescued by NAD^+^ precursors (NAM or NR), demonstrating a direct link between NAD^+^ availability, glycolytic competence, and ATP production during infection. Conversely, in some oncolytic virus contexts, NAMPT inhibition enhances viral cytotoxicity by sensitizing tumor cells to metabolic stress (e.g., reovirus in multiple myeloma models), highlighting that NAD^+^ metabolism can differentially influence viral replication versus oncolysis depending on cellular context.

Our finding that inhibition of glutaminolysis by BPTES suppresses VSV replication further supports the notion that glutamine metabolism provides an important metabolic backbone for infection. Many viruses, including both DNA and RNA viruses, increase glutamine uptake and catabolism to replenish TCA cycle intermediates and support nucleotide, lipid, and amino acid synthesis. For example, Marek’s disease virus relies on glutamine-driven anaplerosis for optimal replication, while white spot syndrome virus in shrimp depends on glucose- and glutamine-fueled de novo nucleotide synthesis [59,60]. In Zika virus-infected cells, transient inhibition of viral protein synthesis has also been observed following blockade of glutaminolysis with BPTES [28]. Together with our data, these studies highlight that glutaminolysis complements glycolysis by sustaining anaplerotic flux and biosynthetic precursor pools necessary for efficient viral replication.

Importantly, our findings emphasize that metabolic pathways such as glycolysis, the PPP, and glutaminolysis are not merely nutrient supply routes, but also tightly intertwined with host antiviral defenses. Viral manipulation of these pathways can therefore profoundly influence infection dynamics, replication efficiency, and cell fate. The metabolic dependencies we identify for VSV—particularly on glycolysis, NAD^+^ homeostasis, and glutaminolysis—mirror broader patterns observed across diverse viral families, including flaviviruses, coronaviruses, and oncolytic viruses [45]. These shared dependencies suggest potential therapeutic windows in which targeted metabolic interventions could be leveraged either to suppress pathogenic viruses or to optimize oncolytic virotherapy in cancer.

From a translational perspective, these findings highlight both opportunities and challenges. Glycolytic inhibitors and glycosylation disruptors such as 2-DG have been explored as antiviral and anticancer agents, but their pleiotropic effects necessitate careful consideration of dosing and context. Likewise, NAD^+^-modulating strategies—including NR supplementation or NAMPT inhibition—can have opposing effects on viral replication and host immunity depending on cellular state and therapeutic goals. While this study focused on A172 cells to allow detailed mechanistic analysis, future studies examining additional glioblastoma models will be important to determine the generalizability of these metabolic dependencies. Importantly, many fundamental metabolic dependencies of viral replication are conserved across cell types, suggesting that the pathways identified here likely represent broader regulatory mechanisms. Furthermore, VSV entry into cells is mediated by clathrin-mediated endocytosis and endosomal acidification [61]. Although we have not directly assessed virus entry and uncoating in our studies presented here, future studies on the effects of the metabolic inhibitors on virus binding, entry, and uncoating would also contribute to the mechanistic understanding of the role of these inhibitors in virus replication processes. A nuanced understanding of how specific metabolic interventions influence both host and viral physiology will therefore be essential for translating metabolic targeting strategies into effective and safe clinical applications, particularly in the context of oncolytic VSV-based therapies.

## 5. Conclusions

In summary, our study demonstrates that VSV replication in glioblastoma cells is tightly regulated by host central carbon metabolism. Pharmacological inhibition of glycolysis and glutaminolysis markedly suppressed viral replication, highlighting VSV’s dependence on metabolic pathways that are characteristically elevated in cancer cells [8,16,44]. Disruption of the PPP revealed that maintenance of cellular redox balance—particularly NAD^+^ availability—rather than nucleotide biosynthesis alone is a critical determinant of productive VSV infection. Consistent with this model, inhibition of NAD^+^ biosynthesis via NAMPT blockade impaired viral replication, whereas supplementation with NAD^+^ precursors restored viral growth, establishing the NAD^+^ salvage pathway as a central metabolic axis supporting VSV replication [7,28].

These findings have important implications for oncolytic virotherapy. Metabolic interventions under investigation for cancer treatment, including glycolytic and glutamine-targeting therapies, may inadvertently constrain oncolytic virus replication or, conversely, be strategically leveraged to enhance tumor selectivity and therapeutic safety [2,13,43]. More broadly, this work underscores how tumor-specific metabolic states shape virus–host interactions and emphasizes the importance of incorporating metabolic context into the rational design of oncolytic virus strategies. Defining the metabolic constraints that govern viral replication will be essential for optimizing the efficacy and safety of cancer virotherapies.

## Data Availability

The original contributions presented in this study are included in the article. Further inquiries can be directed to the corresponding authors.
